# Protocol for a randomised phase 3 trial evaluating the role of Finasteride in Active Surveillance for men with low and intermediate-risk prostate cancer: the FINESSE Study

**DOI:** 10.1136/bmjopen-2024-096431

**Published:** 2025-02-11

**Authors:** Marcus Cumberbatch, Bernard North, Roseann Kealy, Samuel Smith, Rachel Hubbard, Steven Kennish, Selina Bhattrai, William Cross, Rohit Chahal, Richard Bryant, Alastair D Lamb, Mohantha Dooldeniya, Simon Faulkner, Peter Sasieni, James Catto

**Affiliations:** 1 Department of Urology, Sheffield Teaching Hospitals NHS Foundation Trust, Sheffield, UK; 2 The University of Sheffield, Sheffield, UK; 3 Queen Mary University of London, London, UK; 4 Leeds Institute of Health Sciences, University of Leeds, Leeds, UK; 5 Department of Medical Imaging, Sheffield Teaching Hospitals NHS Foundation Trust, Sheffield, UK; 6 Department of Histopathology, Leeds Teaching Hospital NHS Foundation Trust, Leeds, UK; 7 Leeds Teaching Hospitals NHS Trust, Leeds, UK; 8 Bradford Teaching Hospitals NHS Foundation Trust, Bradford, UK; 9 Department of Surgical Sciences, University of Oxford Nuffield, Oxford, UK; 10 Urology, Churchill Hospital, Oxford, Oxfordshire, UK; 11 Department of Urology, Mid Yorkshire Teaching NHS Trust, Wakefield, UK; 12 Metro Charity, London, UK; 13 Division of Clinical Medicine, University of Sheffield, Sheffield, UK

**Keywords:** Prostate, Prostatic Neoplasms, SURGERY, Urological tumours

## Abstract

**Background:**

Prostate cancer (PCa) is the most common male malignancy in the western world. Many men (40%) are diagnosed with localised low or intermediate-risk PCa, which is suitable for active surveillance (AS). AS affords careful monitoring to identify changes in otherwise non-life-threatening cancers. While AS reduces overtreatment (and quality of life impact), long-term compliance can be poor, with many men undergoing radical treatment after starting AS.

**Methods and analysis:**

Finasteride in Active Surveillance for men with low and intermediate-risk prostate cancer (FINESSE) is a prospective, open-label, two-arm, phase 3 trial, in which men with low or intermediate PCa are randomised (1:1) to receive AS with or without finasteride (5 mg once a day for 2 years). Randomisation is stratified by age and PCa risk. AS includes regular prostate-specific antigen testing, MRI scans and the offer of repeat biopsy (at 3 years, or if imaging suggests progression). Additional MRI scans and/or biopsies will be performed for biochemical or clinical indications. We aim to recruit 550 men (aged 50 to 75 years) from up to eight sites. Active outpatient follow-up will be for 3–5 years (depending on date recruited), followed by passive registry-based follow-up for up to 10 years. Primary outcome is adherence to AS. Secondary outcomes include rates and type of disease progression, treatments received (for PCa and benign prostatic enlargement), overall and PCa-specific mortality, an understanding of patients/professionals views of this approach and health-related quality of life. An external panel of experts blinded to allocation will review all AS cessation and progression events. Trial pathologist’s and radiologist’s, blinded to allocation, will review representative cases. Analysis is Intention to Treat.

**Ethics and dissemination:**

The study received Health Research Authority and South-Central Oxford Research Ethics Committee (14/12/2021: 21/SC/0349) and CTA/MHRA (29/12/2021: 21304/0274/001–0001) approvals. Results will be made available to providers and researchers via publicly accessible scientific journals.

**Trial registration number:**

ISRCTN16867955

**Strengths and limitations of this study**

- While active surveillance (AS) is an established method of managing men with prostate cancer, few studies have attempted to improve compliance with this regimen.
- Finasteride is widely available, has a known safety profile, is well tolerated and is used in a similar patient population for benign prostate enlargement.
- This study will determine AS outcomes in a contemporary cohort of intermediate-risk cancers.
- There remains some scepticism about the role of pharmacological prostate-specific antigen manipulation for AS patients.
- Pre-biopsy MRI may reduce the pool of eligible men and hamper recruitment.

## Introduction

 Prostate cancer (PCa) is the most common male malignancy in the western world.^1^ Prostate-specific antigen (PSA) screening of asymptomatic men has been used to reduce mortality from the disease. However, most men diagnosed through this route have clinically localised disease and may not benefit from treatment as their cancers are indolent, with a long natural history, or metastatic at diagnosis.^2^ There has yet to be a universally accepted screening programme for PCa and most men are diagnosed through ‘case-finding’ using PSA testing for lower urinary tract symptoms or known risk factors (eg, family history). The detection and radical treatment of PCa that would not impact the patient during their lifetime represents overdiagnosis and overtreatment, respectively.^3^ One solution to overtreatment is the use of active surveillance (AS).^4^ This strategy selects men with indolent appearing cancers and monitors tumour growth. Radical treatment is reserved for men whose tumours progress biochemically, clinically or radiologically.

In men with low-risk PCa undergoing AS, the risk of disease-specific mortality is small (eg, 0.3% at 8 years and lower than that from competing diseases^5^). AS is popular among men with localised PCa^6 7^ and recommended by the National Institute for Clinical Excellence (NICE) guidelines in the United Kingdom (https://www.nice.org.uk/guidance/ng131). However, there are concerns regarding the accuracy of PCa risk stratification and the reliability of monitoring tools.^8^,^10^ Clinicians and patients fear that deferring radical treatment could reduce the chance of cure and lead to higher morbidity.^10 11^

Between 50% and 70% of men starting AS will receive either radical or palliative treatment over the following 10 years.^12^,^14^ In most men, radical treatment is initiated due to either a rising PSA or changes in Gleason grade on biopsy. Both are surrogate measures for disease progression. Many men are reluctant to undergo multiple biopsies and so most AS programmes are heavily reliant on PSA kinetics. For example, 25% of men in the Gothenberg screening trial^14^ and 43% of men in the Toronto trial who started AS received radical treatment due to a rising PSA alone.^4^ PSA values reflect benign enlargement and inflammation within the prostate^13^ as well as cancer growth. Therefore, many men with rising PSA values may not have disease progression and may not need radical treatment. For example, 65% of men within the PRIAS study^13^ and 72% in a large US series^15^ had favourable histology at radical prostatectomy after a period of AS. Within the ProtecT RCT, 50% of men randomised to monitoring received radical treatment with a <2% mortality rate at 10 years,^12^ highlighting the potential for overtreatment.

Various approaches have been tried to improve compliance with AS, including pharmacological interventions. The REDEEM study group randomised 302 men with low-risk PCa to 0.5 mg daily Dutasteride or placebo.^16^ At 3 years, the Dutasteride group had 10% fewer men with disease progression (defined as increasing cancer burden on biopsy or undergoing radical treatment). The ENACT study group randomised 227 men with low or intermediate-risk PCa to AS with or without 160 mg daily Enzalutamide.^17^ The addition of Enzalutamide reduced progression (pathological or therapeutic) by 46% at 12 months, although no difference was present at 2 years, there were side effects with this agent and its cost poses financial challenges to healthcare providers (especially if for long term AS regimens).

Contemporary AS cohorts include many men with intermediate-risk PCa, as MRI may have changed the spectrum of PCa’s diagnosed. Many men with small, low risk PCas are often no longer diagnosed either because they do not have a biopsy or there is less random prostate sampling.^18^,^20^ Within the PRECISION trial, 38% of men with mpMRI-guided biopsy (vs 24% in ultrasound scan (USS)-guided biopsies) had Gleason 3+4 PCa.^18^ Van der Leest *et al* found that mpMRI-guided biopsy reduced the rate of insignificant PCa diagnosis from 25% to 14%.^19^ Therefore, the focus to improve the care of men with PCa is shifting to using AS in men with intermediate-risk PCa.^21^,^26^ This population is common and includes more men with lethal cancer than in the low-risk cohorts.^5^ Thus, AS regimens need to combine safety with tolerability and adherence. Improving AS was the highest research priority selected in the recent NICE guidelines for PCa management (Question number 1: What is the most suitable surveillance protocol? (https://www.nice.org.uk/guidance/ng131). Given the positive signals from the REDEEM and ENACT trials, this study aims to test if the drug Finasteride can increase men’s adherence to AS and reduce radical treatment rates, using a more contemporary cohort.

## Methods and analysis

### Design

Finasteride in Active Surveillance for men with low and intermediate-risk prostate cancer (FINESSE) is a randomised, prospective, non-blinded, open-label, parallel group, phase 3 trial. Men will be randomised 1:1 to receive AS plus finasteride (5 mg) for 2 years or AS alone.

### Randomisation and population

Randomisation is through a web-based tool bespoke to the King’s Clinical Trials Unit. Once participants have completed a signed consent form (online supplemental file 1), for example, Finesse Consent form, V.5.0 from 27 March 2024, their data will be stored on the system. The randomisation process is at the individual level using the method of permuted block randomisation with block sizes stratified by PCa risk (low vs intermediate) and participant age (<65 vs >65 years).

### Blinding

This is an open label study. Both participants and clinicians will be aware of the study arm to which they are randomised. While test results, for example, MRI scans and PSA values can make it obvious that a participant is taking finasteride, the following will be blinded (not informed) to treatment allocation:

1. Lead Trial Radiologist responsible for reviewing MRI scans.
2. Lead Trial Pathologist responsible for reviewing histopathology.
3. Independent PCa Progression Review Panel, made up of three urologists.

### Study setting

The FINESSE trial is recruiting in secondary care sites. The trial is funded by Yorkshire Cancer Research, a charity whose remit is to fund research which will save lives in Yorkshire, and so initial sites have been established within the Yorkshire region. Non-Yorkshire centres will be included to expedite recruitment. Eligible patients are identified by secondary care clinicians (urologist) in outpatient clinics and multidisciplinary team meetings. Research nurses will support the screening, consent and follow-up processes.

### Recruitment

We aim to recruit 550 men over 24 months. The trial management group will monitor this in real-time and recommend action if recruitment is behind projections (such as opening additional sites, extending recruitment duration or adjusting eligibility (eg, removing biopsy restrictions, increasing the time since diagnosis)). Patient and public involvement in research (PPI) representatives and behavioural scientists will be involved from the outset to ensure that the research questions and study design are relevant to the needs of patients with PCa, to inform the patient facing literature, and to facilitate effective recruitment. Patients may self-refer by contacting their local FINESSE investigator. Informed consent will be obtained by recruiting physicians (onlinesupplemental files 1^2^).

### Eligibility criteria

1. Male subjects aged 50–75 years, with an estimated life expectancy of 10 years or more, who have opted for AS as their preferred PCa management option.
2. Willing and able to provide written informed consent, or if appropriate, have an acceptable individual capable of giving consent on their behalf.
3. Fit enough and suitable for radical treatment.
4. Eastern Oncology Performance status ≤1.
5. A histological diagnosis of Gleason grade group ≤2 (ie, Gleason grade 3+3=6 or 3+4=7) PCa within the last 6 months.
6. Radiological stage ≤T2 b cN0 cM0 as defined by mpMRI imaging within the last 6 months (from the date of the mpMRI scan to the date of the patient’s randomisation). A copy of the mpMRI scan and report-confirming eligibility will be required.
7. PSA ≤20 ng/mL. The result must be within 3 months of the date of the patient’s randomisation.
8. PSA density ≤0.2 ng/mL/mL. The result must be within 3 months of the date of the patient’s randomisation.
9. Biopsy criteria (via either transrectal or transperineal routes) within the last 6 months of the patient’s randomisation date):
  - If targeted biopsy, then the maximum cancer core length is ≤10 mm
  - If targeted and systematic sampling biopsy, then the maximum cancer core length should be ≤10 mm, and ≤2 or ≤15% of non-targeted cores involved with cancer.
  - If non-targeted biopsy (ie, USS template or sampling irrespective of lesions), then maximum cancer core length is ≤10 mm AND ≤3 or ≤20% of total number of cores involved with cancer.

### Ineligibility criteria

1. Previously received treatment for PCa (including radiotherapy, hormone therapy, brachytherapy or surgery). Of note, men who have received treatment for benign prostate enlargement are eligible.
2. Current or recent (≤12 months) treatment with finasteride or dutasteride.
3. Currently enrolled or has been a participant within the last 30 days, in any other investigational drug or device study.
4. Men not willing to comply with the procedural requirements of this protocol.
5. Known allergy/sensitivity to or intolerance of finasteride or dutasteride.
6. Known allergy to any excipients of finasteride.
7. Any malignancy (other than non-melanoma skin cancer and/or PCa) that has not been in complete remission for 5 years.
8. Any serious coexistent medical condition that would make repeat prostate biopsy hazardous.
9. All contraindications to finasteride including concomitant therapy with any medication that may interact with finasteride.
10. Any rare hereditary problems of galactose intolerance, total lactase deficiency or glucose–galactose malabsorption.
11. Men trying for a baby or with a pregnant partner.
12. High-risk disease.

### Usual care: AS

**Figure 1:**
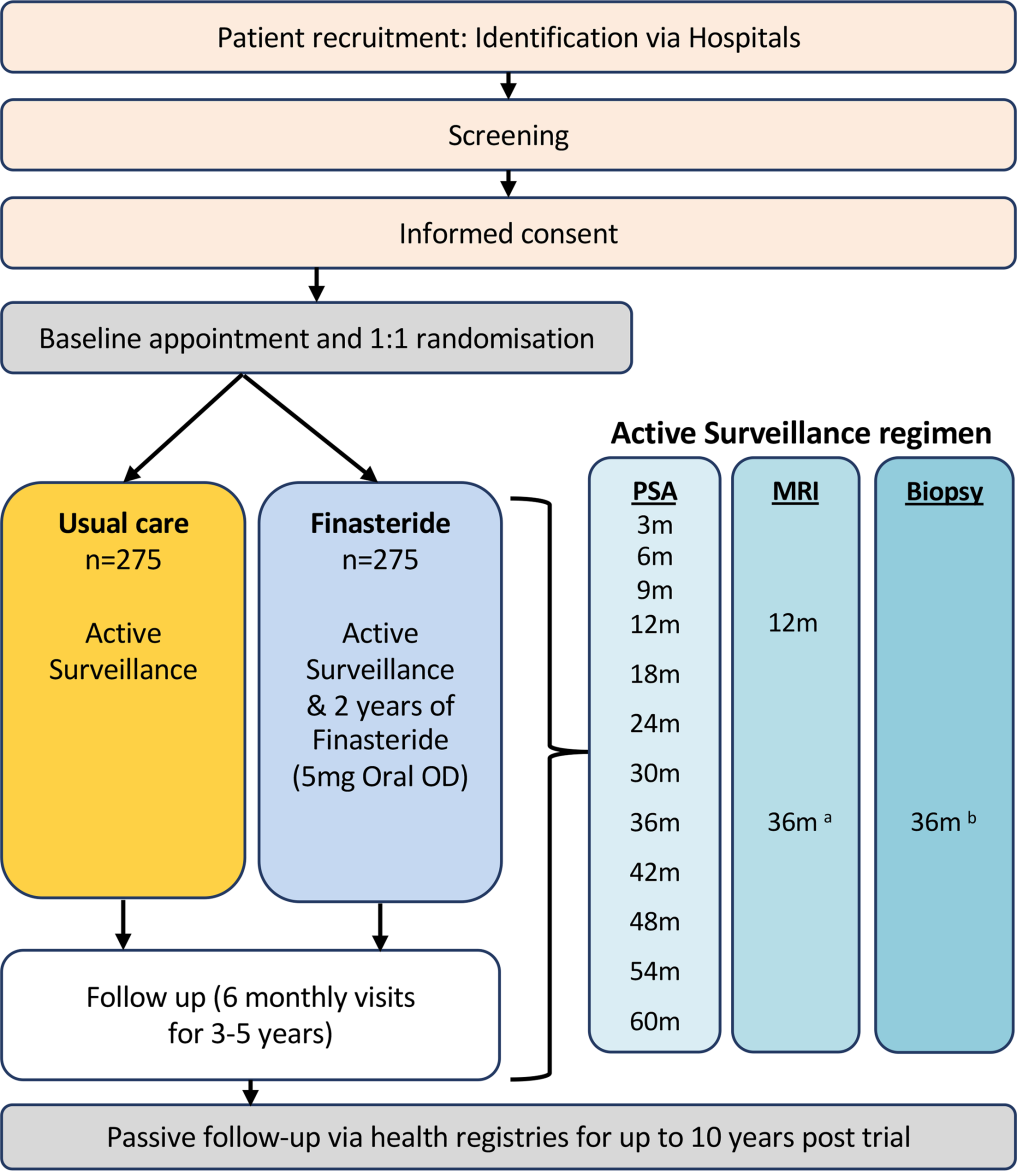
Recruitment and participant flow within the Finasteride in Active Surveillance for men with low and intermediate-risk prostate cancer (FINESSE) study. Follow-up within active surveillance includes PSA testing, MRI scans and the offer of a repeat biopsy (times in months (m) shown). ^a.^ Strongly recommended. ^b.^ Offered as a routine to all men. Also strongly recommended for changing MRI appearance and/or where indicated by the MRI scan. PSA, prostate-specific antigen.

Men randomised to usual care will receive AS (see figure 1). Patients will not receive a placebo, as PSA and MRI changes make masking impossible, blinding PSA data would be impractical since men may actively seek PSA tests outside the study, it is ethical that control participants experiencing any side effects, for example, erectile dysfunction, know they are independent of the treatment, participants unaware they are taking finasteride may opt for radical treatment earlier, and placebo controlled trials are expensive. Concerns regarding PSA changes or digital rectal examination changes will lead to MRI scans outside the schedule. Changes in MRI and PSA will lead to either a rebiopsy (to detail histological grade) or radical treatment. Radical treatment without radiological or pathological evidence of progression is discouraged but not prohibited.

### Finasteride plus AS

Men randomised to the intervention group will receive finasteride (oral 5 mg) to be taken once a day for 2 years, in addition to AS (as above). Participants will be prescribed finasteride on a 3 monthly basis and this will be dispensed from their recruiting hospital pharmacy. Compliance will be measured using pill counts and patient questionnaires.

## Study aims

1. To understand whether the addition of finasteride to AS increases adherence in men with low/intermediate-risk PCa.
2. To understand the tolerability and compliance with finasteride within an AS regimen.
3. To understand whether the addition of finasteride to AS reduces disease progression in these men.

## Objectives and outcomes

The primary and secondary objectives, with matching outcomes, are detailed in table 1 and box 1. We will also detail health-related quality of life, over time, using validated Patient Reported Outcome tools, including decision regret and conflict findings (table 2).

**Table 1:** Primary objectives and outcomes within the FINESSE trial

Objectives	Outcome measures	Timepoint(s) of evaluation of this outcome measure (if applicable)	Additional information
**Primary objective:**To compare adherence with AS in men with low or intermediate PCa with and without 2 years of finasteride during follow-up of between 3 and 5 years from randomisation.Adherence is defined as men who have received neither radical nor palliative treatment, and have remained under surveillance, at each timepoint.	Rate of either radical prostatectomy, radical radiotherapy, brachytherapy or prostate-cancer targeted treatment. Rate of use of systemic therapies. Rate of use of androgen deprivation therapy. Rate of other treatment for PCa. Rate of participant death from PCa. Rate of men discontinuing AS for any other reason.	All cessation from AS events from participants during follow-up of between 3 and 5 years from randomisation, will be included in the first analysis. Later analysis will use passive follow-up (up to 10 years after trial closure).	Rates in each arm will be measured by patient self-reporting. Participants who are lost to follow-up, or who die of a cause unrelated to PCa will be taken as censored.

ASactive surveillancePCaprostate cancer

Box 1 **Secondary objectives and outcomes within the Finesse trial.**

**Outcome measures**

Time until cessation of AS due to initiation of:

- ADT and/or chemotherapy.
- Radical prostatectomy.
- Radical radiotherapy.

**Timepoint(s) of evaluation of this outcome measure (if applicable):**

All occurrences of cessation of AS events due to (1) ADT initiation, chemotherapy, (2) radical prostatectomy (3) radical radiotherapy, (4) other treatment including watchful waiting and (5) death from prostate cancer during participant follow-up, 4 years on average, will be included in the analysis.

The listed reasons for AS cessation will be treated as competing events. Cumulative incidence plots will be presented with a curve for overall AS cessation and for cessation for the individual post-AS treatment.

1. To measure prostate cancer progression.

**Outcome measures**

Progression is defined as either:

- Increase in MRI stage from T2a to ≥T2 c, T2b to ≥T2 c, or T2x to ≥T3 b.^27^
- Increase in grade from gleason 3+3 to ≥3+4 or 3+4 to ≥4+3.
- Radical Prostatectomy histology revealing grade ≥4+3 or stage ≥T3 a.
- PSA progression defined as ≥25% increase from the highest prerandomisation PSA value.
- Radiological confirmation of metastatic prostate cancer including identification via bone and/or PSMA PET scans.
- Clinical record of cancer progression.
- Clinical record of the initiation of palliative care.
- Death from prostate cancer.
- Clinical digital rectal examination (DRE) deterioration*.
- Extraprostatic disease.

(note *DRE results alone will not be considered a definitive endpoint).

**Timepoint(s) of evaluation of this outcome measure (if applicable):**

1. To measure PCa mortality.

**Outcome measures**

Participant death from PCa.

**Timepoint(s) of evaluation of this outcome measure (if applicable):**

All deaths from PCa occurring during the 3–5-year follow-up of the study will be analysed.

1. To study the changes in MRI appearances of the prostate over time in men with/without finasteride.

**Outcome measures**

bpMRI/mpMRI scan results at baseline (the diagnostic MRI), 12 and 36 months (please note, a 36-month MRI scan is strongly recommended).

**Timepoint(s) of evaluation of this outcome measure (if applicable):**

Baseline, 12 and 36 months.

**Additional information:**

We will record:

- Prostate volume from (height, width, length).
- PCa stage: using the Prostate Imaging Reporting and Data System (V.2) and Tumour, Nodes, Metastasis staging.
- PCa size: taken as the maximum diameter on an axial slice from the MRI acquisitions.

The pMRI/mpMRI images will be quality controlled centrally by the Lead radiologist. Full details can be found in the FINESSE Radiology Manual.

1. To understand the views of patients and healthcare professionals regarding the use of finasteride within AS for this disease.

**Outcome measures**

Semistructured one-to-one interviews led by a trained interviewer, with selected individuals during the follow-up phase.

**Timepoint(s) of evaluation of this outcome measure (if applicable):**

Months 48– 60

1. To measure the rate of intervention for symptoms related to benign prostate enlargement:

Defined as the use of oral medication (such as alpha blocker, PDE5 inhibitor or anti-cholinergic) or endoscopic prostate surgery (such as Transurethral resection of the prostate (TURP), Urolift, Green light laser TURP, steam treatment, Holmiun Laser Encucleation of the Prostate (HOLEP) or similar).

**Outcome measures**

Patient self-reporting.

**Timepoint(s) of evaluation of this outcome measure (if applicable):**

All symptoms during the follow-up of between 3 and 5 years until trial end.

**Additional information:**

Determined from new prescriptions for oral medication (such as alpha blocker, PDE5 inhibitor or anti-cholinergic) or the participant undergoing a prostate surgery for benign enlargement (such as TURP, Urolift, Green light laser TURP, steam treatment, HOLEP or similar).

1. Overall (all cause) mortality.

**Outcome measures**

Death electronic Case Report Forms (eCRF) completed by sites.

**Timepoint(s) of evaluation of this outcome measure (if applicable):**

All deaths during the follow-up of between 3 and 5 years until trial ends.

**Additional information:**

Cause of death will be decided by note review (and CRF completion) and death certificates.

**Table 2:** Schedule of events for quality-of-life measures (collected through eCRFs (electronic Case Report Forms)) during the FINESSE trial

		Treatment phase (years 1–2)									Follow-up phase (years 3–5)						
		Timepoint in months (visit can be±2 weeks).															
	Completed by participants on FINESSE web-based EDC, (REDCap)	Randomisation	3	6	9	12	15	18	21	24	30	36	42	48	54	60	Early withdrawal
Quality of life measures	EQ-5D-5L	x	x	x		x		x		x		x		x		x	x *†
	EORTC QLQ C30	x	x	x		x		x		x		x		x		x	x *†
	EPIC	x	x	x		x		x		x		x		x		x	x*†
	EORTC QLQ FA12	x	x	x		x		x		x		x		x		x	x*†
	Memorial Anxiety Scale Prostate Cancer	x	x	x		x		x		x		x		x		x	x*†
	Depression Anxiety Stress Scales (DASS) 21	x	x	x		x		x		x		x		x		x	x*†
Decision-making measures	Decisional Conflict Scale	x				x				x		x		x		x	x*†
	Subjective Decision Quality	x				x				x		x		x		x	x*†
	Decisional Regret	x				x				x		x		x		x	x*†
	Decisional Involvement	x				x				x		x		x		x	x*†
Adherence	Voils DOSE-Non adherence measure		x	x	x	x	x	x	x	x							x ‡

* Where a participant stops treatment and/or trial participation early, due to radical treatment, they will continue to receive these questionnaires for completion, for the remainder of their intended period of follow-up, providing they consent to do so. The exception for this group is the ‘Decisional Conflict Scale’ which will not be assessed again, and the decisional involvement scale which will only be administered once more, post radical therapy.

† Where a participant stops treatment and/or trial participation early, for any reason other than radical treatment, they will continue to receive these questionnaires for completion, for the remainder of their intended period of follow-up, providing they consent to do so.

‡ If the participant is still on treatment at the point of early withdrawal, one final Voils DOSE-Nonadherence measure—Extent Scale will be sent for completion.

## Sample size

We estimate finasteride will reduce AS cessation rates by 50% (from 20% to 10%) after an average of 4 years follow-up. The sample size of 550 men (275 perm arm) is based on a time to event analysis with 90% power to reject H0: HR≠1, that is, the detection of a significant difference in AS cessation rates between arms by use of a two-sided log-rank test with alpha=0.05. We assume that 50% of control participants will progress (or be treated) during follow-up and that the HR is 0.65. The exact number needed is 271 per arm. We believe we will need to screen 1500 men to obtain 550 eligible, consenting recruits.

## Statistical methods

### Participant population

The main endpoint analysis of progression from AS will be performed on all participants who have been randomised on an intention-to-treat basis. For the log-rank and Cox proportional hazards assessment of time to AS progression, the assumption of proportional hazards between the AS and control arms will be conducted by plotting log cumulative hazard plots. Kaplan-Meier plots will be produced to both aid the comparison of time to AS between treatment arms and to assess violation of the non-proportional hazard assumption. A formal assessment of proportional hazards will be performed by cumulative martingale residual plots with p value assessment of the Brownian bridge property present when proportional hazards is approximately satisfied. In the event of the occurrence of a significant degree of non-proportional hazards, then we will compare groups using Schemper’s weighted model. The analysis of Quality of Life (QOL) questionnaires will be performed on the set of men who complete the questionnaires. Tolerability of finasteride analysis will be performed on all participants randomised to finasteride.

### Procedure(s) to account for missing or spurious data

We anticipate that the dropout level will be low. For the main endpoint of progression from AS participants who withdraw from the trial or who are lost to follow-up will be censored at the last attended visit or the time of notification of withdrawal.

### Premature termination of the trial

There is no intention to perform an interim analysis to stop on grounds of efficacy. Although there are no safety concerns related to finasteride, the IDMC (Independant data monitoring committee) will review safety data produced by the trial statistician and have the power to recommend termination on that basis.

### Other statistical considerations

Any deviations from the statistical analysis plan will require justification to the IDMC and approval by the TSC.

## PCa progression panel

Some of the progression events in PCa or reasons for cessation of AS can be open to investigator bias. Given that this trial is open label, to minimise bias and inform broader clinician agreement regarding progression, an independent panel of urologists will review each case of progression or AS cessation. Members of this panel were selected based on recognition of their expertise in managing PCa and knowledge of AS. The panel will agree to the presence (or absence) of progression and classification (eg, radiological, pathological, biochemical). It was considered optimal to have a panel that is independent of the NHS.

## Data collection, monitoring and harms

Three systems will be used to collect data for the FINESSE trial:

1. The randomisation system: used to randomise participants and allocate a personal identification number (PIN).
2. The FINESSE electronic data capture system (EDC), referred to as simply the EDC within the protocol): a web-based EDC system designed, using the InferMed Macro 4 system for collection screening log information, trial electronic Case Report Forms (eCRF) and generating prescriptions.
3. REDCAP: used to collect patient identifiable data, participant surveys, Pateint reported outcome measures (PROMs), and registry data.

Several methods will be implemented to maximise data completeness. The Finesse EDC has in-built validation checks to alert for missing or unusual data. There will also be manual reviews where data monitoring queries can be raised. There will be league tables for posting metrics on completeness of data from each site. Finally, there will be automated phone Short Text Messages and email reminders to participants to optimise Quality of Life questionnary completion.

A formal risk assessment has been undertaken for the trial to identify and propose mitigation strategies for the main risks to ensure safe and successful delivery of the trial. A list of these risks is explained in greater detail in the FINESSE Risk Assessment Log. The risk assessment has defined the FINESSE study as moderate risk and as such, monitoring of the trial will be conducted using a risk-based approach following the monitoring plan developed by the trial team.

A combination of onsite, remote and central monitoring will be undertaken, to an agreed frequency and schedule. The interval for monitoring visits may be longer or shorter, dependant on subject enrolment rates, quality issues, trial site compliance, other trial site issues or any event(s) that affect the overall conduct of the study. The trial DM/Monitor will arrange a date and time with the appropriate person and site staff to ensure documents are available for the visit. Sites will be given at least 2 weeks’ notice of any monitoring visit. The site principle investigator (PI) will be met at each visit, where possible.

## Ethics and dissemination

### Approval, protocol amendments, consent

The study received approval from the Health Research Authority and South-Central Oxford Research Ethics Committee (14 December 2021: 21/SC/0349) and CTA/MHRA (29/12/2021: 21304/0274/001–0001). The study is sponsored by Sheffield Teaching Hospitals NHS Foundation Trust. The sponsors have no role in the collection, interpretation or dissemination of the trial findings. The protocol will be submitted by those delegated to do so, to the relevant Research and Development department of each participating centre. A copy of the local Confirmation of Capacity and Capability and of the Patient Information Sheet and Consent Form, on local headed paper should be forwarded to the Cancer Prevention Trials Unit (CPTU) before participants are entered. An agreement will be in place between each centre and the CPTU setting out respective roles and responsibilities.

Approval for release of Hospital Episode Statistics (HES) data and access to data processed by the National Cancer Registration and Analysis Service will be obtained from NHS Digital or replacement body at the time of application. The Trial Master File will hold all approvals and relevant communications with the aforementioned bodies and be maintained by the CPTU.

Informed consent will be obtained prior to randomisation (online supplemental file 1, Finesse Consent form).

Results will be made available to providers and researchers via publicly accessible scientific journals and presentations at academic meetings. Results will be shared with patient groups through the funders (Yorkshire Cancer Research) and relevant patient groups.

### Confidentiality and access to data

The investigator(s)/site(s) will permit trial-related monitoring, audits, REC review and regulatory inspection(s), providing direct access to source data and documents. Study participants will be informed of this during the informed consent discussion. The process will include participants being asked to consent to provide access to their medical notes and/or to any online registries that contain information related to their diagnosis. Access to data will be limited to the minimum number of individuals necessary for quality control, audit and analysis.

### Amendments to protocol since recruitment started

Several amendments to the protocol have been completed since the initial protocol and the trial opened to recruitment. Please see these detailed in online supplemental appendix 1.

## Trial status

The trial opened to recruitment in August 2022 with the first participant randomised at St. James’s University Hospital, Leeds on the 23 September. The study is in the active recruitment phase.

## supplementary material

10.1136/bmjopen-2024-096431 online supplemental file 1

10.1136/bmjopen-2024-096431 online supplemental file 2

10.1136/bmjopen-2024-096431 online supplemental file 3
