# Supplementary material for: Protocol for a randomised phase 3 trial evaluating the role of Finasteride in Active Surveillance for men with low and intermediate-risk prostate cancer: the FINESSE Study
Source: BMJ Open. 2025 Feb 11;15(2):e096431. doi: 10.1136/bmjopen-2024-096431 (PMC11815428; doi:10.1136/bmjopen-2024-096431)
Supplement: online supplemental file 1 [file bmjopen-15-2-s001.docx]

**Appendix 1: Amendment History**

| **Amendment No.** | **Protocol version no.** | **Date issued** | **Author(s) of changes** | **Details of changes made** |
| --- | --- | --- | --- | --- |
| 1). (Non- substantial) | 2.0 | 03.03.2022 | Roseann Kealy | Administrative changes to the protocol to reflect a shift of responsibility to the study team for some of the functionality originally assigned to the MACRO Electronic Data Capture (EDC) system. The build of the latter is being outsourced and the vendor in question was unable to support all the features we had anticipated. Changes include:  • Centrally monitoring the number of participants allocated to the low and intermediate-risk groups to ensure set quotas are observed. Previously this was being managed by the application.   - Sites to keep local screening logs outside of the EDC. - Some electronic case report form names have been changed to align with the vendor’s nomenclature system.   • Removal of the provision of a back-up randomisation system. The treatment is not urgent, and we have been informed outages are very rare.  • If e-consent is required, e.g., in the event of a pandemic, this will now be in REDCap, not MACRO.   - Prescriptions will no longer be printed by the application. - SAE reporting and data collection for the MRI & Pathology Central Reviews are now being conducted outside of MACRO.   • The data flow diagram (xii) and appendices 5a & 5b summarising the eCRFS completed by site staff on MACRO and REDCap respectively, have been updated to reflect the above.  • Removal of the self-referral process for patients contacting the FINESSE CCO directly. |

| **Amendment No.** | **Protocol version no.** | **Date issued** | **Author(s) of changes** | **Details of changes made** |
| --- | --- | --- | --- | --- |
| 2). (Substantial) | 3.0 | 18.05.2022 | Roseann Kealy | The following administrative changes have been made to the protocol: • Amendment of the term ‘transgender women’ to ‘transgender persons’.  •  References for the qualitative assessment tools being used in the trial have been added to section 15 of the protocol.  •  The IMP destruction policy has been clarified.  •  Units added to PSA density  •  All text stating no data will be transferred outside of the UK has been amended, since TWILIO, the third party we are using to send SMS reminders to participants on our behalf, has servers based in the US and Europe. No REDCap data is ever stored on the Twilio servers. REDCap requires disabling Twilio’s Request Inspector. The Request Inspector is a tool provided by Twilio that lists all requests made between Twilio and an external application. When configuring Twilio for a REDCap project, REDCap checks that the Request Inspector is disabled before enabling Twilio for the project.  •  Details regarding the issuing of the Participant Identification Number (PIN) have been clarified, in particular which system generates it - EDC MACRO, not the Randomisation System.  •  Further detail regarding the transfer of patient identifiable information.  •  Revision of the pathology review process. It will be the responsibility of the FINESSE CCO to monitor pathology reporting discrepancies at site. Should the Lead Pathologist record a higher rate of disagreement than expected, this will be discussed with the TMG, who may consider increasing the proportion of biopsies to be centrally reviewed. |

| **Amendment No.** | **Protocol version no.** | **Date issued** | **Author(s) of changes** | **Details of changes made** |
| --- | --- | --- | --- | --- |
| 3). (Substantial) | 4.0 | 19.06.2023 | Roseann Kealy | The following significant changes have been made to the protocol:  •  Eligibility criteria updated to increase the time since PCa diagnosis from 6 months to 2 years and participants' last MRI scan from 6 months to 12 months.  •  Inclusion and explanation of the ‘Hub and Spoke Model’ (HSM), in the protocol and PIS, to augment the use of district general hospitals (DGHs)  as PICs, with the potential to conduct standard of care procedures (SoC), and those within usual care competence (WUCC), for the FINESSE trial. DGHs will act as ‘spoke’ trial sites to the ‘hub’ investigator site. It will also allow for reduced patient burden (i.e., complete transfer to the hub) incorporating patient choice. The HSM will be used in accordance with the HRA Integrated Research Guidelines.  •  The addition of Pinderfields Hospital, Mid Yorkshire Hospitals NHS Trust as a site.  •  Clarification that it is also the sites' responsibility to check the completed patient quality of life questionnaires in the FINESSE Study database for adverse events and serious adverse events.  •  Minor changes to the wording of the primary and some secondary objectives to make them clearer.  •  The addition of an outcome measure 'Rates of participant death from PCa’, to the primary objective.  •  Separation of the quality-of-life objectives from the secondary objectives and recategorised as explanatory objectives, to reflect their role more accurately. These explanatory objectives provide further context to the primary and secondary objectives relating to adherence.  •  Anonymisation of the central pathology and radiology review process.  •  Correction to the location of the Data Safe Haven (DSH).  •  Clarification that MRI reports only, not scans, may be sent from NHS-to- NHS email instead of via IEP.  •  Change of Principal investigator at Oxford. Mr Richard Bryant will be replacing Mr Alastair Lamb.  •  Clarification of the sample size calculation wording.  •  Removal of the maximum threshold value of 33% of low-risk participants across all sites. The recruitment rate is lower than anticipated, and we do not wish to restrict it further.  **The following non-significant changes have also been made to the protocol:**  •  Typo of age eligibility criteria on page 39 corrected to <65 years.  •  Clarification that participants will be asked to return their unused medication every 3 months including the 18 months timepoint which was erroneously missed form the following list: 3, 6, 9, 12-, 15-, 21- & 24- month time points.  •  Clarification that for radiological stage, MX will be treated as M0, and NX as N0 in this study.  •  Updates to the contact details of Data Monitoring Committee member, Dr Sam Merriel, who has changed institutions.  •  Clarification that bpMRI scans will be accepted instead of mpMRI scans when determining radiological disease stage, to accommodate sites not conducting multi parametric scans.  Finally, the following two additional new documents are also being submitted:  •  A new patient information sheet addendum to be used with the PIS at hub and spoke sites explaining the Hub and Spoke model.  •  A new version of the ICF to cover the hub and spoke model. |
| 4). (Non-substantial) | 4.0 | 27.09.2023 | Roseann Kealy | The following changes have been made to the protocol to address three sections where updates to the timelines were missed within the recently approved substantial amendment:   - The trial summary table states, "Men aged 50 to 75 years diagnosed with low/intermediate-risk localised prostate cancer in the 6 months preceding their date of randomisation".   This has been corrected to, "Men aged 50 to 75 years diagnosed with low/intermediate-risk localised prostate cancer in the 24 months preceding their date of randomisation".   - Section 5.1 states, “Eligible men aged 50 – 75 years with low or intermediate-risk prostate cancer diagnosed within the last 6 months will be invited to join the trial”.   This has been corrected to read, Eligible men aged 50 – 75 years with low or intermediate-risk prostate cancer diagnosed within the last 24 months will be invited to join the trial”.   - Section 6.1b states “Prior active surveillance populations: Recruiting hospitals can assess their databases to identify potentially eligible patients already managed by active surveillance, diagnosed within the last 6 months.”   This has been corrected to read, “Prior active surveillance populations: Recruiting hospitals can assess their databases to identify potentially eligible patients already managed by active surveillance, diagnosed within the last 24 months.” |
| Substantial | 5.0 | 17. 10.24 | Harriet Strachan & Roseann Kealy | - Change of institution of the Cancer Prevention Trials Unit from King’s College London to Queen Mary University of London. - Change of institution for Peter Sasieni (Co- Lead Applicant & Trial Statistician), Bernard North (Independent Trial Statistician) and Roseann Kealy (FINESSE Study Trial Manager) from King’s College London to Queen Mary University of London. - Update to indemnity section to add Queen Mary University of London. - Clarification:   - That the secure restricted access server Data Safe Haven maintained by a contracted GDPR compliant third-party storage provider that stores patient identifiable data for the study will now be retained by King’s College London and Queen Mary University of London.   - Of the IMP destruction policy.   - Of the requirements for a valid PSA density result.   - Of the 'outcome measures' for the secondary objective 'To compare between Finasteride with AS and AS alone, the rates of cessation of AS due to initiation of ....' - Removal of the limit on number of cores and maximum cancer core length, from inclusion criterion 10, to increase the pool of potentially eligible men. - Additional advice for Investigators and research staff to improve awareness of the potential risk of psychiatric and sexual dysfunction side effects, and to highlight the need to monitor these closely, within section 7.10. |
