# Supplementary material for: Protocol for a randomised phase 3 trial evaluating the role of Finasteride in Active Surveillance for men with low and intermediate-risk prostate cancer: the FINESSE Study
Source: BMJ Open. 2025 Feb 11;15(2):e096431. doi: 10.1136/bmjopen-2024-096431 (PMC11815428; doi:10.1136/bmjopen-2024-096431)
Supplement: online supplemental file 2 [file bmjopen-15-2-s002.pdf]

[FINESSE STUDY LOGO]

[Please print on local headed paper & add contact details of the local research

CONSENT FORM

FINESSE: ***A medical research study to improve treatment for men with early prostate cancer***

Name of Principal Investigator:

**PIN:**							

Please **initial** box

1. I confirm that I have read and understood Parts 1 and 2 of the Participant Information Sheets, Version no: X Dated: DD/MM/YYYY for the above study. I have had the opportunity to ask questions, and these have been answered to my satisfaction. I understand how to raise a concern or make a complaint. ☐

2. I understand that my participation is voluntary, and that I am free to withdraw from the study at any time, without giving a reason, and without my medical care or legal rights being affected. ☐

3. I understand that relevant extracts from my medical notes, data and tissue collected, may be looked at by the clinical trials unit co-ordinating this research, researchers from the Universities of Sheffield and Leeds, the Sponsor, Sheffield Teaching Hospital NHS Trust, and also by the regulatory authorities or from the NHS Trust, where it is relevant to my taking part in this research. I give permission for these individuals to have access to my records. ☐

4. I understand that even if I withdraw from the study, the information and samples collected from me up to that point will be used in the analysis of the results, and my identity will remain anonymous within this analysis. ☐

5. I understand that my name and contact details will be collected and securely stored on a secure, restricted access server Data Safe Haven maintained by a contracted GDPR compliant third-party storage provider based within the UK, who are retained by Kings College London or Queen Mary University of London. My details will be used to send me relevant information relating to the study, to track my health long-term via relevant health data registries e.g. The National Cancer Registration and Analysis Service (NCRAS), and to request additional information relevant to the trial, from local health information sources, e.g., my treating hospital. ☐
   As detailed in the PIS, I am aware that employees of third-party providers, based outside of the UK, and contracted by the research team, may require access to my personal-identifiable data to fulfil their role as a third-party service provider. However, my personal-identifiable data will be kept strictly confidential and never be stored outside of the UK.

6. I understand that where relevant, slides of tissue collected for standard care biopsies & corresponding pathology reports, may be sent from my hospital's Pathology Department to the Pathology Department at Leeds Teaching Hospital NHS Foundation Trust, for central review by the FINESSE Lead pathologist. Slides & reports will be pseudo-anonymised, and the pathology samples will be returned to the sites once the review is complete, and in accordance with the site's pathology release conditions. ☐

[Please add contact details of the local research team]

7. I understand that pseudonymised and anonymised data generated from the Trial may be made publicly available and shared with commercial/overseas researchers within Europe or organisations, to support other research in the future, and may be shared anonymously with other researchers or organisations which may include those in the commercial sector, here or within Europe. ☐

8. I agree to update the FINESSE Coordinating Centre of any relevant changes to my personal details e.g., a change to my email address, or a new telephone number. ☐

9. I agree to my General Practitioner being informed of my participation in the study. ☐

10. I agree to take part in the above study. ☐

**Optional interview consent**	*Please* ***initial*** *relevant box below*	
1. I agree to be approached by the research team and invited to participate in a 60-minute telephone interview exploring my experience of taking part in this study. I understand that my participation in the interview is voluntary and that I am free to withdraw at any time without giving any reason.	Yes	
	No	

**Optional future contact consent**	*Please* ***initial*** *relevant box below*	
2. I agree to be contacted about future studies using the contact details I have provided.	Yes	
	No	

Name of Participant: *Date:* *Signature:*

Name of Person taking consent if not PI: *Date:* *Signature:*

Name of PI/Delegated Investigator: *Date:* *Signature/Countersignature:*

*1 copy for the participant; 1 copy for Investigator Site File (ISF); 1 (original) to be kept in medical notes.
