# Supplementary material for: Protocol for a randomised phase 3 trial evaluating the role of Finasteride in Active Surveillance for men with low and intermediate-risk prostate cancer: the FINESSE Study
Source: BMJ Open. 2025 Feb 11;15(2):e096431. doi: 10.1136/bmjopen-2024-096431 (PMC11815428; doi:10.1136/bmjopen-2024-096431)
Supplement: online supplemental file 3 [file bmjopen-15-2-s003.pdf]

[Please print on local headed paper and add contact details of the local research team & Trust Logo]

Short Title: FINESSE – A research study to improve treatment for men with early prostate cancer

Scientific Title: The FINESSE Study: A randomised phase 3 trial evaluating the role of Finasteride in increasing compliance with active surveillance, in men with a new diagnosis of low and intermediate risk prostate cancer, when compared with usual care.

You are being invited to take part in the FINESSE study. This is a clinical trial for men diagnosed with prostate cancer. To help you decide whether to take part you need to understand why the research is being carried out and what it would involve.

For the purposes of this information sheet, the term 'we' refers to the Cancer Prevention Trials Unit at King's College London, who are responsible for co-ordinating and running this study on behalf of the Sponsor and the Chief Investigator. Please see section 21.

If this information sheet and consent form contain words you do not understand, please ask the study doctor or nurse to explain anything unclear. Please take time to read the information carefully. You will be able to take a copy of this sheet home so you can read it again. If you want to, you can discuss it with family or friends before deciding. If you choose not to take part, your healthcare will not be affected.

You should not sign the consent form until you have read this information sheet carefully, asked any questions you might have, and received satisfactory answers.

Part 1

1. What is the purpose of this study?

This trial will try to find out if a drug called finasteride can support men to continue with active surveillance after they have been diagnosed with localised prostate cancer. If this works, it will increase the number of men who avoid or delay the need for further treatment, and the side-effects accompanying this.

One of the popular treatment options for low or intermediate risk prostate cancer is active surveillance. Active surveillance means, rather than treating you with surgery or radiotherapy, your doctor will monitor you for signs that your cancer is changing. That way you would only need further treatment if you and

your doctor agree you do. Active surveillance is used because some prostate cancers never progress beyond the stage they are at when they are found, and so do not need further treatment.

Once active surveillance begins, you'll have regular tests to check on the cancer. One of the tests is a prostate specific antigen (PSA) test. This test measures the amount of PSA in your blood. PSA is produced by normal cells in the prostate and also by prostate cancer cells. A raised PSA level may suggest a problem in your prostate, but not necessarily cancer. PSA tests can be unreliable and can suggest prostate cancer is present when no cancer exists. They can also incorrectly indicate that a man does not have prostate cancer when they in fact do. PSA levels in men with prostate cancer can vary and can go up even when cancer is not progressing. Most men with low or intermediate risk prostate cancer do not require further treatment, but higher PSA levels may make men worry and this is a common reason why men decide to have further treatment.

We aim to improve what is offered for men like you so that you feel more confident in safely staying on active surveillance, using a drug called finasteride. Finasteride is used to improve symptoms of enlarged prostates, but also reduces PSA levels. We think that reducing PSA levels with finasteride might help your clinician to assess your prostate cancer more accurately by stopping it from rising due to factors that are not related to your prostate cancer (such as inflammation or normal enlargement associated with ageing). The decision regarding the need for further treatment will be more focused on the results of a prostate biopsy and prostate MRI, rather than fluctuating PSA levels. However, PSA levels will still be considered by your doctor because if your cancer is progressing, they can still rise, even if you are taking finasteride.

This is a randomised controlled trial, which means if you take part, you will be allocated to one of two study arms chosen at random. You and your medical team cannot choose which group you are put into. Half of the men will be placed into the active surveillance AND finasteride group (intervention arm) and half into active surveillance ONLY (control arm). We will recruit 550 men and allocate them to these groups. You and your doctor will both know which group you are in. This is what we call an 'open label' study. The study will run for five years, but if you are randomised to the intervention arm you will only take finasteride tablets for two of those years. Please note, if you are randomised to the control arm but are then prescribed finasteride for another medical reason by your treating clinician or GP, you will have to be withdrawn from the study.

2. What does taking part in this study involve?

Men who choose active surveillance for further treatment are seen regularly in a hospital clinic. Most of the time, the clinic and research appointments will be at the same time. However, participants will be required to attend up to three additional appointments as part of the trial, including a consent and randomisation visit at the start and two additional visits during the second year of the study. You can claim up to £25 per visit for your travel expenses to attend these extra visits. Please note, if current pandemic

policies change during the study, your study appointments may happen by telephone or video call, and your study drug if you are randomised to the finasteride arm may be posted to you.

Consent and randomisation visit – consent and random allocation to the study group can take place on the same day for men who have had a PSA test done in the last 3 months.

First year of the study – Local staff have been asked to ensure as far as possible, that research appointments coincide with regular active surveillance appointments, so that no extra study appointments are required during this period. Men allocated to the treatment arm will also take one tablet of finasteride (5mg) every day during this year.

Second year of the study - during the second year of active surveillance, men are usually seen every 6 months in their regular active surveillance Clinic. Finesse study visits will continue to be scheduled every 3 months so participants will be asked to attend **two extra visits, one at month 15** and the other **at month 21,** during this period. Men in the treatment arm will continue to take one tablet of finasteride (5mg) every day during this year. The treatment will be stopped after two years.

Third, fourth and fifth year of the study - men will continue to attend routine active surveillance appointments every 6 months and all study appointments will take place at the same time. Men allocated to the treatment arm will no longer take finasteride tablets during this period.

All men in the trial will be asked to complete questionnaires approximately every three months, which should take between 20 and 30 minutes in total to complete. This is to check how you are getting on, as we want to keep track of how your health and treatment may affect your quality of life.

These questionnaires will be emailed to you in between your visits. For this reason, we will collect your personal contact details, with your permission.

Some men will be invited to take part in a telephone interview at the end of the trial. Questions in this interview will relate to their experiences of taking part in the trial. The interview is optional.

Flow Diagram of Order of Events:

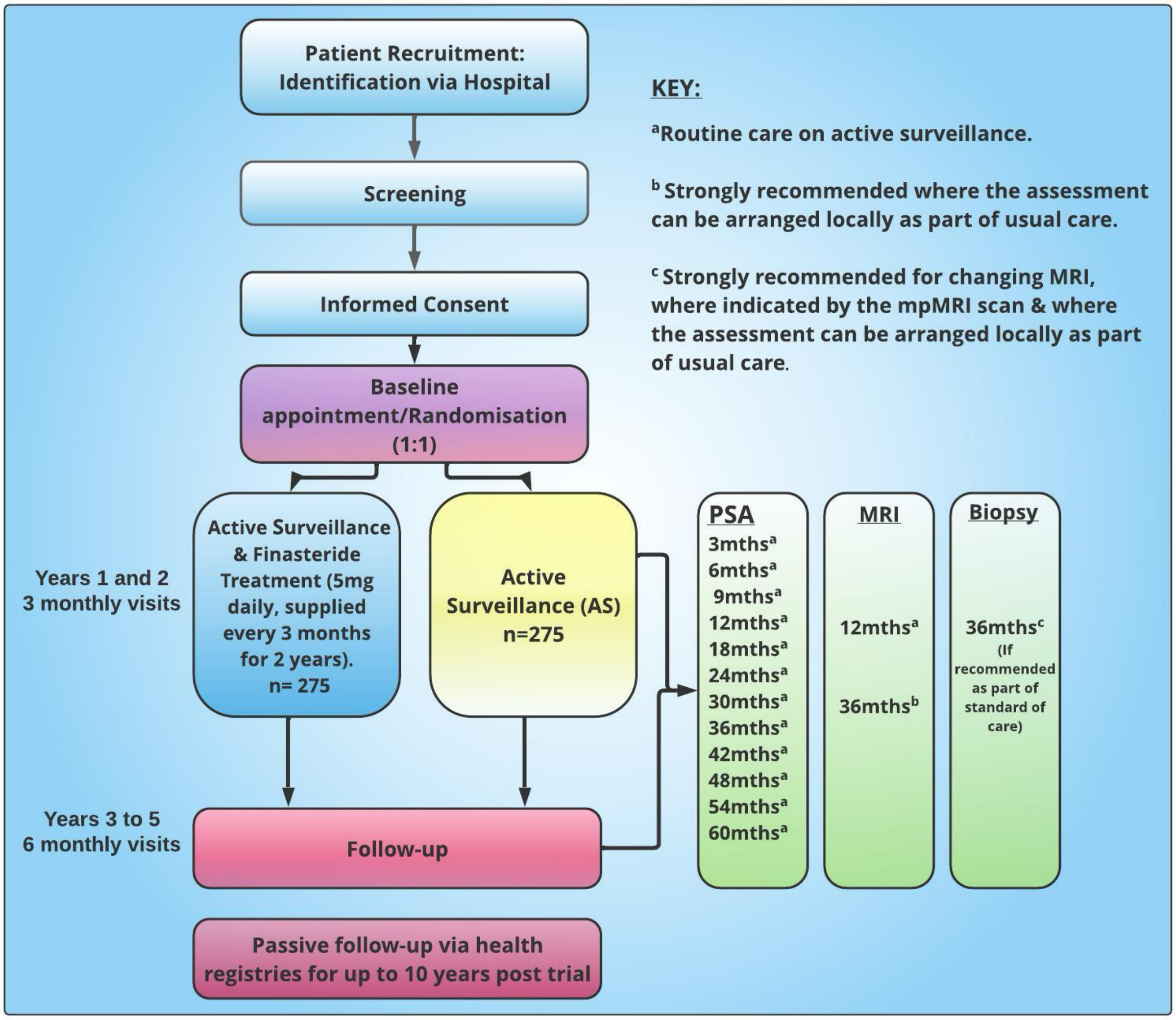

3. Why have I been invited?

You are being invited to take part in this study because your doctor believes your type of prostate cancer and treatment makes you suitable.

You **are potentially able** to take part in this trial if you:	You are **not able** to take part in this trial if you:<br><br>• have previously received treatment for prostate cancer

• have been diagnosed with prostate cancer in the last 12 months<br>• have not received previous treatment for prostate cancer<br>• have opted for active surveillance for prostate cancer<br>• are fit and suitable for radical treatment<br>• are aged 50-75 years old at diagnosis	• are currently taking or have been taking finasteride or dutasteride in the last 12 months<br>• you are planning to father a child<br>• you have been told you have a terminal illness

There are some additional eligibility criteria related to your diagnosis and other medical conditions you may have. A research nurse will ask you questions in person at the clinic, or over the phone, and look at your medical records, to check that you are suitable.

If you are interested in the trial, but unsure whether you can take part, please contact your research nurse (contact details on the front page).

4. I am transgender or a non-binary person, can I still take part?

Yes. Whilst the terms 'men' and 'male' are used throughout the study documents, the trial is open to anyone with prostate cancer regardless of gender (including transgender /non-binary persons), providing they satisfy the inclusion and exclusion criteria).

5. Do I have to take part if I am suitable?

No. It is completely up to you whether you take part or not. If you do not wish to take part, your healthcare will not be affected in any way. If you do decide to take part, you will be asked to read and sign a consent form. Even if you consent to taking part in this trial, you can change your mind and leave the study at any time, without giving a reason.

6. What is the medicine being tested?

The medicine being tested is finasteride. Finasteride will be in tablet form, 5mg in a single tablet, taken once a day. Ideally this will be around the same time every day, with water. The tablet will be coated to avoid irritating the stomach lining. All men in the treatment arm will be asked to take finasteride for 2 years.

Finasteride is also known by the brand names Proscar and Propecia. It is a type of medicine called a 5-alpha reductase inhibitor which works by stopping testosterone (a sex hormone) turning into another hormone called dihydrotestosterone (DHT), which can cause your prostate to grow bigger. Finasteride

stops DHT being produced which helps shrink your prostate. It is therefore used to treat men with an enlarged prostate (benign prostate enlargement). It can help ease symptoms such as frequent and urgent urination, difficulty completely emptying the bladder or starting urination. Some studies have suggested that it **MAY** shrink the prostate tumour, but this is not the main objective of the FINESSE study.

In this study, finasteride is being used 'off label' which means the medicine is being used in a way that is different to that described in the licence.

7. Are there any alternative treatment to the study?

For men who have already made the decision to join an active surveillance programme, your alternative to this study is not to take part. There are alternatives to active surveillance which your doctor will have discussed with you, including surgery. However, if you are unsure what those alternatives are, or you would like to discuss them again, please ask your doctor, who will talk you through them in detail.

8. What are the possible side effects of taking part?

Like all medicines, finasteride can cause side effects, but not everyone will get them. Finasteride is well tolerated and does not normally cause serious side effects.

Common Side Effects (happen in more than 1 in 100 people):

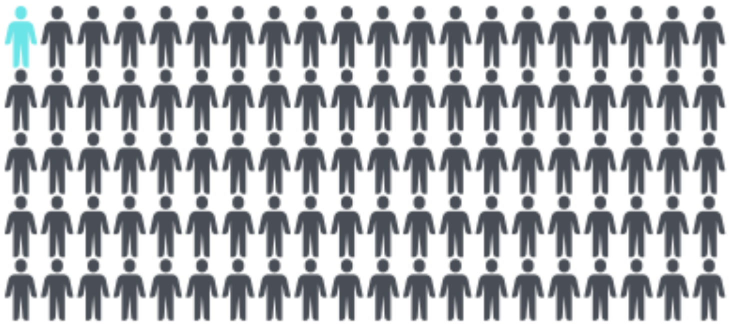

These usually improve after a while, but they should be discussed with a doctor if they bother you or do not go away:

- less interest in having sex (decreased libido/sex drive)
- trouble getting or keeping an erection.
- problems with ejaculating, such as little or no semen

- increase in breast size and tenderness.
- skin rash

Serious side effects:

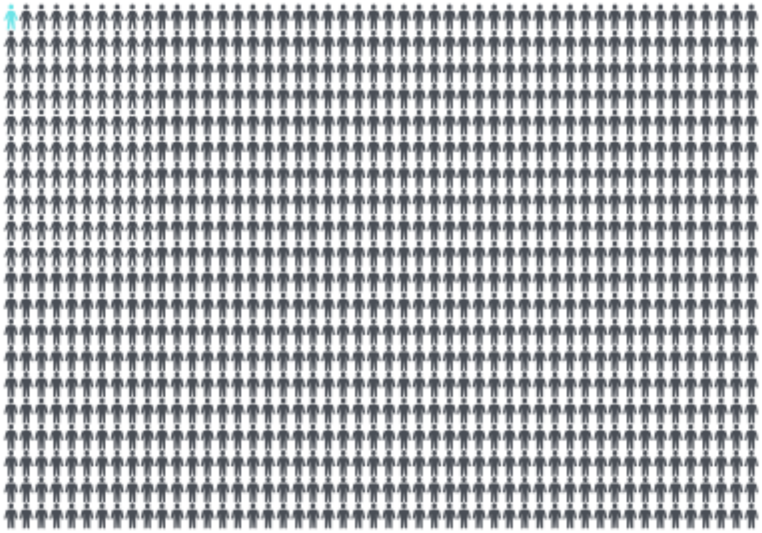

Serious side effects are rare and happen in less than 1 in 1,000 people. Some people may notice these side effects after taking finasteride for a few months. These should always be reported to a doctor.

- Lumps, pain or swelling in your chest area or discharge from your nipples
- Unusually low mood (depression) or thoughts of harming yourself
- Allergic reaction- in rare cases, finasteride may cause a serious allergic reaction (anaphylaxis), in which case immediate action such as calling 999 or going to A&E, would be required

A full list of side effects will be provided inside the medicine packet.

Special note on pregnancy:

Even though finasteride is not generally prescribed for women, and no women will be recruited into the FINESSE trial, it could still harm an unborn baby. Therefore:

1) Men trying for a baby or with a pregnant partner will not be allowed to take part in the trial.
2) Participants taking finasteride will be advised to:
   a. Use a condom when having sex. This is because small amounts of finasteride pass into semen.

b. Inform their partners not to touch any crushed or broken finasteride tablets if there's any chance they could be pregnant. Finasteride can get into your bloodstream through your skin if you handle **broken** tablets. This is why the tablets come with a protective coating.

A pregnant partner of a male trial participant taking finasteride, will be followed up as per standard clinical care, i.e., by the gynaecology/midwifery/paediatric team caring for the pregnant partner. Participants and/or their partners are advised to notify the team the male partner is/has been taking finasteride.

A child born to the partner of a male trial participant taking finasteride, will be followed up as per standard clinical care, i.e., by the gynaecology/midwifery/paediatric team caring for the neonate/infant. Participants and/or their partners are advised to notify the team the male partner is/has been taking finasteride.

9. What are the other possible disadvantages and risks of taking part?

If you have already chosen to have active surveillance a disadvantage is that some of your appointments might take a little longer than normal. You will need to remember to take a tablet every day, unless advised otherwise. Completing the online questionnaires may also take some time.

10. What are possible benefits of taking part?

- You may avoid or delay more intensive treatment for prostate cancer, which may have benefits for your quality of life.
- The growth of your prostate cancer **MAY** be slowed down (with the drug), although further research is needed to see if this is definitely true, and this is not the main objective of the FINESSE study.
- You may help improve the care of men with prostate cancer who opt for active surveillance and help us better manage the disease e.g., by promoting the use of other technologies in active surveillance such as MRI scans.
- If you have benign disease in addition to prostate cancer, you may see improvements in this.
- You will have more regular follow-ups than is standard practice.

11. What happens at the end of the trial?

The study will run for five years (two years to cover the treatment period, and three years of follow-up). Men taking finasteride will be asked to stop taking the drug after two years. The trial is not funded to offer

finasteride treatment after two years. Because the drug is being used ‘off-label’ in this study, you will need to consult your urologist if you wish to continue taking finasteride after your two years’ trial treatment.

Men whose prostate cancers have remained unchanged at that point will continue to be followed up as part of the normal active surveillance programme. In addition, during years 3, 4 and 5 of the study, you will continue to complete study questionnaires every 12 months.

If at any stage your cancer shows signs of change and you need further treatment, your doctor will advise you to stop the study treatment. You can still complete the study questionnaires.

With your permission, once you have finished your trial appointments, the research team will continue to collect information from your doctor or from central NHS records for up to ten years to track your health, including whether you have received further treatment for prostate cancer. This is sometimes called ‘Passive follow-up’ because it takes place without requiring any involvement from study participants. If you do not want this to happen, you can say you want to stop any more information being collected.

At the end of the trial, your data will be stored securely and used to answer our research questions. The findings from the trial may be reported at meetings, conferences, and published in journals in a way that no-one can work out who took part in the study. More information on the storage and use of your data can be found on in section 23. Data handling and confidentiality.

12. What if something goes wrong?

You should contact your doctor or nurse if you have a question or a problem while taking part in the research. Their contact details can be found in section 13 of this information sheet. If you are seen by a doctor outside the study, you should remind them you are taking part in FINESSE. In case of emergency, you should act in the same way you would if you were not on the study. It is unlikely that you will need emergency hospital treatment as a result of this trial. However, you should always inform any doctor treating you that you are taking finasteride 5mg.

The overall sponsor of the trial is the Sheffield Teaching Hospitals NHS Foundations Trust (STHNHSFT), and the trial is coordinated by the Cancer Prevention Trials Unit at King’s College London (KCL).

NHS indemnity will provide cover for negligent harm relating to STHNHSFT’s role as trial sponsor. As employers of the authors, KCL and the University of Sheffield (UoS) provide indemnity to cover negligence only liabilities arising from the design of the research. You may be able to claim compensation if you can prove that STH NHS, KCL and/or the University of Sheffield has been negligent.

However, as this clinical trial is being carried out in hospital, the hospital continues to have a duty of care to you. STHNHSFT, KCL & UoS do not accept liability for any breach in the hospital's duty of care, or any negligence on the part of hospital employees. This applies whether the hospital is an NHS Trust or

otherwise. In the case of NHS sites, NHS indemnity will provide cover for negligent harm occurring from the conduct of the trial at NHS sites.

If you sustain injury as a result of negligence and wish to make a claim for compensation, you should do so in writing in the first instance to the Chief Investigator via the CPTU. Address details can be found on the trial website. This will then be passed to the relevant insurer. Hospitals participating in the FINESSE Study must provide clinical negligence insurance cover for harm caused by their employees and a copy of the relevant insurance policy or summary should be provided upon request.

No arrangements for payment of compensation in the event of harm to the research participants where no legal liability arises have been made by the Sponsor.

13. Will my taking part in the study be confidential?

Yes. All the information about your participation in the study will be kept confidential. Further details about this can be found in Part 2.

14. Who should I call if I have questions, queries and/or complaints?

- You can ask more questions about the study at any time, and you can contact the following people for more information: 'local PI name' and 'research study nurse' – the study doctor and research nurse

  Telephone: [Sites to enter local number]

- You can also visit the FINESSE study website at: www.finessetrial.org

- For independent advice on taking part in a clinical trial please contact 'local' Health Patient Advice and Liaisons Service (PALS) on Sites to enter local PALS number] or email: [Sites to enter local PALS email]

  The PALS service is available [Sites to add local PALS opening hours]

- If you want to complain about how researchers have handled your information, you should contact the research team. If you are not happy after that, you can contact the Data Protection Officer on info-compliance@kcl.ac.uk

If you are not happy with their response or believe they are processing your data in a way that is not right or lawful, you can complain to the Information Commissioner's Office (ICO) (www.ico.org.uk or 0303 123 1113).

This completes part 1 of the Information Sheet.

If the information in Part 1 has interested you and you are considering taking part, please continue to read the additional information in Part 2 before making any decision.

Part 2

15. What should I do if I want to take part?

If you have received this leaflet from one of the urological clinics at participating NHS centres please contact the research nurse working on the trial (see section 13).

If you have found this Patient Information Sheet on the FINESSE website, or elsewhere on the Internet, please register your interest by emailing finesse@kcl.ac.uk

If you are interested in taking part, the next steps include the research nurse:

- Checking that you are suitable (if they have not already done so), by asking you a series of questions about your health
- Booking a consent and randomisation visit. During this visit you will be asked to complete a consent form indicating that you understand what the trial involves and that you agree to take part. Once all of these have been completed, you will then be randomised to one of two groups.

Please only agree to take part in this study if you are willing to accept allocation to either group. Participation in both groups is important to help us find out whether finasteride can reduce the number of men who receive radical treatment for prostate cancer.

16. What if new information becomes available?

Sometimes, during a research study, new or important information about the medicine(s) being studied becomes available. If this were to happen, the trial staff would let you know and discuss it with you. Depending on what the information is, you may wish to withdraw from the study, or your doctor may advise you to withdraw. If you withdraw you would continue to be seen in the normal active surveillance clinics. If you decided to continue in the study, you may be asked to sign an updated consent form.

A special group of experts, known as a Data Monitoring Committee, who are independent from the trial staff and doctors, has been set up to oversee the study on a regular basis to make sure any issues are looked into properly and that the men taking part are informed about any relevant new information. The information sheet and other study documents will also be updated with any new details.

17. What will happen if I don't want to carry on in the study?

You are completely free to leave the study any time you wish and for any reason. The standard of care you receive will not be affected. However, the more men we have on the study, the more data we collect, and the better our chances of answering our research questions accurately. Your participation is important to us and valued. Therefore, we would encourage you to talk to us before making your final decision, to see if we can address any problems that you may be having and improve your trial experience.

If you change your mind about taking part in the study, you can withdraw at one of three levels:

1. It is possible for you to stop the study medication (finasteride), and remain in the study, under follow-up clinic, or by telephone. In this case, you will be asked to continue completing the study questionnaires. During follow-up, and for up to ten years after the trial has finished, the research team will continue to collect some information from central NHS records to track your health, in particular if you had received further treatment for prostate cancer during that period. This type of follow-up is often called 'passive follow-up' because trial participants are not actively involved or inconvenienced.
2. You can decide to stop the study medication (finasteride) AND stop completing the study questionnaires. During follow-up, and for up to ten years after the trial has finished, the research team will access central NHS records to check if you had received further treatment for prostate cancer during that period, (passive follow-up).
3. Alternatively, you may wish to withdraw from ALL aspects of trial. In this case, you will stop taking study medication (finasteride), we will stop sending you the study questionnaires and we will not access national health registries to check if you had received further treatment for prostate cancer during the follow-up period, (passive follow-up).

Information and samples that have been collected up to the point of your withdrawal will remain part of the study. We need to manage your records in specific ways for the research to be reliable. This means that we won't be able to let you see or change the data we hold about you. Research could go wrong if data is removed or changed.

Please ask the study doctor or nurse if you have any questions about this.

18. Will my taking part in the study be confidential?

In this study, most of the research team will not need to know your name. In these cases, someone will remove your name from the research data and replace it with a code number. This is called coded data, or the technical term is pseudonymised data. For example, your blood test might be labelled with your

code number instead of your name. It can be matched up with the rest of the data relating to you by the code number. In the FINESSE study, this code number is called a Patient Identification Number (PIN). Your PIN will be used on most of your study records and samples instead of your name, wherever possible, to ensure information is kept confidential. Please see 'Who will have access to my data?' on page 18 for the exceptions.

Your medical records may be looked at by people who are authorised to check that the study is being carried out properly, and the quality of the research. Representatives of health regulatory authorities and the hospital NHS Trust, and auditors from the Trials Unit and Sponsor may have access to your medical records, and these people will be required to keep your information confidential. A responsible representative from King's College London will also require access to records for the purpose of monitoring and auditing. By signing the consent form you are giving your permission for this to happen.

Your contact details and information collected about you will be stored on a secure database, and access will only be available to members of the trial team, other members of King's College London who may wish to monitor the study, and a third party based outside of the UK who will send text messages on our behalf. These details will also be required to send you study related information questionnaires, and to allow the study team to collect registry data during passive follow-up. Your personal identifiable data will never be stored outside of the UK.

19. Information for your General Practitioner

By signing the consent form, you give the study doctor permission to inform your family doctor (GP) that you will be taking part in this research. We feel that it is important because your GP should be aware of any treatment or medications that you receive so they have a more complete picture of your health. After you have joined the study, they will receive a letter that will include information about finasteride (if you are in the group taking it) and this information sheet for their records. We also encourage you to mention this trial the next time you see your GP.

20. Will any genetic tests be done?

No. We may decide to collect additional samples for testing in the future, but if this were to happen, the trial staff would let you know and discuss it with you. You would also be provided with an updated Patient Information Sheet. You may also be asked to sign an updated consent form.

21. What will happen to the results of this study?

It will take up to 5 years to complete this study, so it will be some time before any results are available. The findings from the trial will be shared with participants and may be reported at meetings, conferences, published in journals and shared with the medical community. If the results from this study are published, your identity will remain confidential and no personal identifiable information will be used.

22. Who is funding the study, and who else is involved?

The research is funded by Yorkshire Cancer Research, and the National Institute for Health Research (NIHR) provides support services within the NHS hospitals involved. The medicines for this trial are being sponsored by the NHS Commissioners.

The Chief Investigator of the study is Professor James Catto (University of Sheffield) and his co-investigator is Professor Peter Sasieni (King's College London).

Sheffield Teaching Hospital NHS Foundation Trust (STHNFT) is organising this research, is the sponsor for the study and employs the Trial Radiologist.

Leeds Teaching Hospital NHS Foundation Trust employs the Trial Pathologist.

The University of Leeds employs the Trial Behavioural Scientist.

The study is being co-ordinated and managed by the Cancer Prevention Trials Unit at King's College London.

None of the staff involved in the study will receive payment specific to their involvement in this research.

22. Data handling and confidentiality

This section outlines how your data will be used, stored, and accessed, during and after the trial.

What is patient data?

When you go to your GP or hospital, the doctors and others looking after you will record information about your health. This will include your health problems, and the tests and treatment you have had. They might want to know about family history, if you smoke or what work you do. All this information that is recorded about you is called patient data or patient information and is also referred to as personal data.
When information about your health care joins together with information that can show who you are (like your name or NHS number) it is called identifiable patient information. It's important to all of us that this identifiable patient information is kept confidential to the patient and the people who need to know relevant bits of that information to look after the patient. There are special rules to keep confidential patient information safe and secure.

Will the use of my data meet UK GDPR rules?

UK GDPR stands for the United Kingdom General Data Protection Regulation. In the UK we follow the UK GDPR rules and have a law called the Data Protection Act. All research using patient data must follow UK laws and rules.

Universities, NHS organisations and companies may use patient data to do research to make health and care better. Universities and the NHS are funded from taxes, and they are expected to do research as part of their job. They still need to be able to prove that they need to use patient data for the research. In legal terms this means that they use patient data as part of 'a task in the public interest'.

If they could do the research without using patient data, they would not be allowed to get your data.

Researchers must show that their research takes account of the views of patients and ordinary members of the public. They must also show how they protect the privacy of the people who take part. An NHS Research Ethics Committee (REC), an independent group of people, checks this before the research starts to protect your interests.

This study has been reviewed and given favourable opinion by the South-Central Oxford C Research Ethics Committee.

King's College London Data Protection Statement

King's College London has a responsibility to keep information collected about you safe and secure, and to ensure the integrity of research data. Specialist teams within King's College London continually assess and ensure that data is held in the most appropriate and secure way. This may include storage of personal data with a contracted GDPR compliant third-party storage provider within the UK, where they are assessed as the best data storage option. Employees of the third parties will have access to your data to fulfil their role as a third-party service providers, but your records and information will be kept strictly confidential.

FINESSE Study Data Protection Statement

Your data will be processed under the terms of UK data protection law [including the UK General Data Protection Regulation (UK GDPR) and the Data Protection Act 2018]. The sponsor, Sheffield Teaching Hospitals NHS Foundations Trust, is the Data Controller and is responsible for looking after your information and using it properly. The KCL Data Protection Officer provides oversight of KCL activities involving the processing of personal data, and can be contacted at info-compliance@kcl.ac.uk

Data protection regulation requires that we state the legal basis for processing information about you. In the case of research, this is 'a task in the public interest.' Special category personal data is personal data that reveals racial or ethnic origin, political opinions, religious or philosophical beliefs, trade union membership, health (the physical or mental), sex life or sexual orientation, genetic or biometric data. The lawful basis used to process *special category personal data* will be for scientific and historical research or statistical purposes.

If you would like more information about how your data will be processed in accordance with UK GDPR, please visit the link below:

https://www.kcl.ac.uk/research/support/research-ethics/kings-college-london-statement-on-use-of-personal-data-in-research

What data will be collected?

In this study we will collect data from five different sources:

1. Clinical data - When you have medical treatment or visit a clinic, a nurse will collect data about your prostate cancer diagnosis and treatment, and other medical conditions which are relevant to the trial. All research should only use the patient data that it really needs to do the research. You can ask what parts of your health records will be looked at.

2. Directly from you - We will ask you to provide your date of birth (DOB), and your NHS and hospital ID numbers to help us locate your MRI scans, pathology, PSA results and other relevant reports. We will also ask you to provide your email address, home address and mobile phone number so that we can contact you and send you online questionnaires. We will always make sure that as few people as possible can see this sort of information that can show who you are.

The online questionnaires will ask about your quality of life, symptoms you experience, your emotional state, and your treatment. If you are in the finasteride group, we will also ask if you took the pill every day and if you are having any issues with taking the drug. All research should only use the patient data that it really needs to do the research. You can ask what parts of your health records will be looked at.

3. Biological tissue - As standard in the NHS, during a prostate biopsy small samples of tissue are taken from the prostate. Once the doctors finish their diagnosis, the trial pathologist may review the biopsy tissue and/or digital images of the tissue where available. This is known as 'central review', and it is carried out to ensure the local radiologists at each of the study sites are reporting results in a similar way. It is a quality control exercise. The comments from their review will not be traceable back to you. With your permission, we will store these images with your study data. You will not be asked to provide any additional prostate tissue for the study. All samples will be managed in accordance with the requirements of the Human Tissue Act (2004).

4. Medical imaging (mpMRI) – You will undergo a diagnostic imaging procedure (magnetic resonance imaging, MRI), as part of your active surveillance, whereby a MRI scanner will scan your prostate. The number of scans you have will be dependent on your individual needs, and is something you will discuss with the urologist treating you. You will not have any additional scans as part of this study. Your doctors will look at the results of this imaging. With your permission, the Trial Radiologist will also centrally review and store these images with your study data. They will be a valuable research resource. As with the pathology samples the comments from the Trial Radiologist's review will not be traceable back to you.

5. Health data registries – This data is protected by data laws and strict access requirements. With your permission we will use these data registries to learn about your long-term health, such as any further treatment you may have for prostate cancer.

If you take part in the additional telephone interview at the end of the trial, your call will be recorded and typed out in a transcript.

Where possible, we will anonymise or pseudonymise the personal data you provide. Pseudonymisation is a technique that replaces information in a data set that identifies an individual, with an artificial identifier. In the case of FINESSE study, this artificial identifier will be a Patient Identification Number or PIN. We will always minimise the processing of personal data wherever possible.

You can find out more about how we use your information at www.hra.nhs.uk/information-about-patients

How will my data be stored?

Prostate tissue samples will be held within Leeds Teaching Hospital NHS Foundation Trust, where the Trial Pathologist is based and mpMRI images will be held by Sheffield Teaching Hospital NHS Foundation Trust (STHNFT), where the Trial Radiologist is based. Digitised images of the tissue samples and copies of the MRI scans will be stored in the Data Safe Haven (DSH). . The DSH will be maintained by AIMES, a contracted GDPR compliant third-party storage provider based within the UK.
It is a secure place we use to store all personal, sensitive, pseudonymised electronically captured data, and other confidential study data, e.g., your questionnaire responses, for access exclusively by approved researchers and clinicians only. In addition, your identifiable patient information will be kept separately from your clinical data.

Who will have access to my data?

Only authorised members of the research team at King's College London will have access to your identifiable data. The exceptions to this are:

Your mobile number, since the third party responsible for sending you reminder text messages is based outside of the UK. The sharing of this data will be in accordance with UK GDPR, and your data will never be stored by this third party.

Your MRI scans and associated reports as it is not possible to anonymise these for this study. However, these will be transferred electronically from one NHS hospital to another NHS hospital using an established and secure transfer process, so the associated risk is considered to be low. We have recommended that where possible, sites use only your NHS number and year of birth to identify you.

Anonymous or pseudonymised data will be viewed by the trial oversight committees and auditors who regulate the trial and ensure everything is done to protect you and your data.

When information is anonymised, it means that data is processed in a manner that makes it impossible to identify individuals from them. Pseudonymisation means that it can no longer be linked to a specific person without the use of additional information. Such additional patient identifiable information (e.g., name and address) must be kept separately from the pseudonymised personal data.

The research nurses/team at your hospital will have access to your medical records to make sure you are suitable for this trial.

Stored, pseudonymised and anonymised data and samples may be used by other researchers, for future medical and health-related research, but only if they have relevant approval from a Research Ethics Committee who look after your interests and ensure the integrity of potential research. Data will not be shared outside of Europe.

How will my data be used?

We will keep all information about you confidential, safe and secure. We will link data about you collected during this trial with other existing health data collected in the UK, such as The National Cancer Registration and Analysis Service (NCRAS). NCRAS collects data on all cases of cancer that occur in people living in England. In order to link your data with other health data, we will use your personal details, such as your NHS number, to link to information in the NCRAS.

Health data collected from any health or social care provider will be securely transferred to the trial team and uploaded onto the trial database. Restricted access to this data will be given to authorised and trained personnel working on the study, and the identifiable personal information will be stored on a secure, restricted access server DSH maintained by AIMES, a contracted GDPR compliant third-party storage provider based within the UK.

Your personal data will be processed so long as it is required for the research project. Researchers from the Sheffield Teaching Hospitals NHS Foundations Trust (STH NHS), Sheffield University, the University of Leeds and King's College London will analyse your data, to see if:

- Taking the drug finasteride results in reduced rates of radical or advanced cancer treatments
- Taking finasteride helps men stay on active surveillance safely, for longer.
- Prostate cancer progresses more slowly in men taking finasteride compared with men not taking finasteride
- Participants have any difficulties sticking to finasteride treatment
- The trial has any impact on participants' wellbeing
- Taking finasteride reduces the number of men receiving treatment for prostate cancer that has spread

The findings will be written up into research papers and published alongside the data, as well as presented at meetings and conferences. However, the reports about the study will be written and presented in a way that no-one can work out that you took part in the study. This personal data will be stored for a minimum of 5 years after the completion of this study in case we need to check it or use it for future research. In addition, the hospital where you are taking part in the study will keep a copy of the research data along with your name. You can ask about the hospital who will keep it, whether it includes your name, and how long they will keep it.

Future Research

If you agree to take part in this research study, you will get the choice for us to keep your contact details and some of your health information, so we can invite you to take part in future clinical trials or other studies. Your data will not be used to sell you anything. It will not be given to other organisations or companies except for research.

Thank you for reading this information leaflet. Should you now decide to proceed with your participation in this study, you will be asked to sign a consent form. Please note that you will be given a copy of this information leaflet and a copy of the signed consent form to keep.
