# Supplementary material for: Protocol for a randomised phase 3 trial evaluating the role of Finasteride in Active Surveillance for men with low and intermediate-risk prostate cancer: the FINESSE Study
Source: BMJ Open. 2025 Feb 11;15(2):e096431. doi: 10.1136/bmjopen-2024-096431 (PMC11815428; doi:10.1136/bmjopen-2024-096431)
Supplement: Supplementary file 4 [file bmjopen-2024-096431.reviewer_comments.pdf]

PEER REVIEW HISTORY

BMJ Open publishes all reviews undertaken for accepted manuscripts. Reviewers are asked to complete a checklist review form and are provided with free text boxes to elaborate on their assessment. These free text comments are reproduced below.

ARTICLE DETAILS

Title (Provisional)

Protocol for a randomised phase 3 trial evaluating the role of Finasteride in Active Surveillance for men with low and intermediate-risk prostate cancer: The FINESSE Study

Authors

Cumberbatch, Marcus; North, Bernard; Kealy, Roseann; Smith, Samuel; Hubbard, Rachel; Kennish, Steven; Bhattrai, Selina; Cross, William; Chahal, Rohit; Bryant, Richard; Lamb, Alastair D.; Dooldeniya, Mohantha; Faulkner, Simon; SASIENI, PETER; Catto, James

---

VERSION 1 - REVIEW

**Reviewer**	**1**
**Name**	**Thakur, Mathew**
**Affiliation**	**Thomas Jefferson University Hospitals**
**Date**	**11-Dec-2024**
**COI**	**I understand and consent to the named publication of this review.**

This reviewer is uncertain as to why the authors wish to publish a protocol rather than its outcome. Only the results of the study will enrich the clinical practice for AS and enhance that database of use of finasteride.

●There are a few typographic errors that should be corrected.

●Line 263: Needs to be clarified

●Line 394:"Trial opened to recruitment in August 2022" but lines 363-365 state "approved will be sought" employing that the protocol approval process is not yet

complete. Please clarify.

●In study Aims, innovation is unclear.

●In table 2, too many secondary objectives are stated. The protocol description does

not assure that all these objectives will be addressed.

**Reviewer**	**2**
**Name**	**Björnebo, Lars**
**Affiliation**	**Department of Medical Epidemiology and Biostatistics, Karolinska Institutet**
**Date**	**02-Jan-2025**
**COI**	**I understand and consent to the named publication of this review.**

Very interesting and important trial! I think it checks all the boxes. My only concern is with the current EMA pharmacovigilance warning regarding finasteride/dutasteride and their potential link to depression and suicide (the current evidence is still rather unclear whether or not there is an association). Should perhaps depression or similar past medical history be part of the exclusion criteria?

VERSION 1 - AUTHOR RESPONSE

Reviewer: 1
Dr. Mathew Thakur, Thomas Jefferson University Hospitals
Comments to the Author:
This reviewer is uncertain as to why the authors wish to publish a protocol rather than its outcome. Only the results of the study will enrich the clinical practice for AS and enhance that database of use of finasteride.
Reply: Thank you. We hope to produce and subsequently report these through this trial. We do believe in the value of publishing protocol papers to improve scientific reporting.

●There are a few typographic errors that should be corrected.
Reply: Thank you. We have screened the paper and corrected all that we have seen.

●Line 263: Needs to be clarified
Reply: Thank you, however, we are unsure what needs clarifying.

●Line 394:"Trial opened to recruitment in August 2022" but lines 363-365 state "approved will be sought" employing that the protocol approval process is not yet complete. Please clarify.
Reply: Thank you for this. This was typo error. We have removed this from lines 363-365.

●In study Aims, innovation is unclear.
Reply: Thank you. To our knowledge, no prior study has tested Finasteride in this context, or in a contemporary population. We hope this is the innovation and that our findings will improve care for these men.

●In table 2, too many secondary objectives are stated. The protocol description does not assure that all these objectives will be addressed.

Reply: Thank you. We agree there are many secondary objectives. The nature of prostate cancer and Active Surveillance means that many possible patient events can happen. We have mapped these out and developed a protocol (with resources) to capture these. We understand the reviewers concerns but do believe these are all relevant.
Please also note Appendix 1 within the paper. You will see that whilst this paper refers to the original version of the protocol, the latter has since been updated to separate the quality-of-life objectives from the secondary objectives and recategorise them as explanatory objectives, to reflect their role more accurately. These explanatory objectives provide further context to the primary and secondary objectives relating to adherence

Reviewer: 2
Dr. Lars Björnebo, Department of Medical Epidemiology and Biostatistics, Karolinska Institutet
Comments to the Author:
Very interesting and important trial! I think it checks all the boxes.
Reply: Thank you.

My only concern is with the current EMA pharmacovigilance warning regarding finasteride/dutasteride and their potential link to depression and suicide (the current evidence is still rather unclear whether or not there is an association). Should perhaps depression or similar past medical history be part of the exclusion criteria?
Reply: Thank you and this is a very relevant question. We have discussed this in the team, spoken with each recruiting centre and have agreed we will continue to recruit men with a history of depression, but with safeguarding strategies in place. We believe there are equal arguments to suggest they may benefit from AS rather than opting for radical treatment (and erectile dysfunction etc. that can further impact mental health). Furthermore, as you say, the current evidence is still rather unclear, and it may be that the data we collect can contribute to the lack of evidence.

Site staff are trained at the site initiation visit to screen for at risk patients and to advise men specifically about depression and suicide during their IMP consultation. In addition, all men complete a series of Quality-of-Life questionnaires including the Depression Anxiety Stress Scales (DASS)-21 [Lee, D., The convergent, discriminant, and nomological validity of the Depression Anxiety Stress Scales-21 (DASS-21). J Affect Disord, 2019. 259: p. 136-142]. every 3 months, and their responses are monitored both locally and centrally.

To date, we have stopped treatment in one man for this concern.

Finally, side effect and pharmacovigilance data is presented regularly at Trial Management Group (TMG)meetings, and periodically to the Data Monitoring and Trial Steering Committees. Neither the TMG nor the committees have any concerns.
